# A Qualitative Risk Assessment of Rabies Reintroduction Into the Rabies Low-Risk Zone of Bhutan

**DOI:** 10.3389/fvets.2020.00366

**Published:** 2020-07-14

**Authors:** Sangay Rinchen, Tenzin Tenzin, David Hall, Susan Cork

**Affiliations:** ^1^Department of Livestock, National Centre for Animal Health, Ministry of Agriculture and Forests, Thimphu, Bhutan; ^2^Department of Ecosystem and Public Health, Faculty of Veterinary Medicine, University of Calgary, Calgary, AB, Canada

**Keywords:** qualitative risk assessment, rabies reintroduction, incursion, risk zone, Bhutan

## Abstract

In Bhutan, dog-mediated rabies has been successfully eliminated from most regions of the country but remains endemic in the Southern region and sporadic incursions are also reported in the East. Elimination of rabies from the southern part of Bhutan is challenged by the porous border with the neighboring states of India which facilitates free and unregulated movement of animals. Around 17 outbreaks of rabies are reported annually in dogs and other domestic animals, posing continuous public health risks and economic losses. Furthermore, due to anthropogenic factors, such as increasing human settlements along highways, increased animal transportation, and the complex and changing human-pet relationship, there is potential to reintroduce rabies from rabies high-risk zone to rabies low-risk zone. This study was undertaken to estimate the risk of rabies re-introduction to the rabies low-risk zone by performing a qualitative risk assessment. The assessment was conducted for three risk pathways (stray dog-pathway, pet dog-pathway and cattle-pathway) under two scenarios: (1) no risk mitigation measures in place and (2) current risk mitigation measures in place. The current control measures include Government led programs, such as mass dog vaccination and dog population management, regulation of the movment of animals through pre-travel check-up and health certification, regular awareness education and rabies surveillance in the rabies endemic areas. The probability of an event occurring was assigned using the data from the available literature. Where gaps in knowledge existed, expert opinion, elicited through modified Delphi method, was used. Under the scenario in which no risk mitigation measures were in place, the risk of rabies re-introduction was estimated to be medium for the stray-dog pathway with a low level of uncertainty, low for pet-dog pathway with a low level of uncertainty, and very low for the cattle-pathway with a medium level of uncertainty. When current risk-mitigation measures were included, the risk of rabies reintroduction was estimated to be very low for the stray-dog pathway with a medium level of uncertainty, low for the pet dog-pathway with a low level of uncertainty, and extremely low for the cattle-pathway with a medium level of uncertainty. The risk of rabies re-introduction through all the pathways was greater than negligible. These findings highlight the importance of maintaining and enhancing current risk mitigation measures to prevent re-introduction of rabies into rabies low-risk zone.

## Introduction

Rabies is a fatal viral disease of mammals, mainly transmitted by dogs. Globally, 59,000 humans die of rabies annually due to dog-mediated rabies and the economic loss associated with the disease is estimated to be USD 8.6 billion (1). In Bhutan, rabies has been successfully eliminated from the northern and central regions through ongoing mass dog vaccination and restricted culling in the early 90s (2). However, rabies remains endemic in parts of the southern region that shares a porous border with the neighboring Indian states of Assam and West Bengal. Occasionally, outbreaks associated with incursion have been reported in two districts of eastern Bhutan that share a border with Arunachal Pradesh, India (3, 4). Rabies incursions occured in three districts of eastern Bhutan in 2005 and two sub districts in south-west Bhutan in 2008 (4, 5). Subsequently, there have been a number of more recent incursions into the South and East but no outbreaks have been reported in the northern and central region since 1991 (2, 6). The outbreaks in eastern Bhutan were associated with the movement of a rabies infected dog from across the Indian border (incursion) while the outbreaks in 2008 occurred due to the movement of a rabid dog from the adjoining rabies high-risk zone in south Bhutan. Dogs are the primary reservoir for rabies in Bhutan. Sustained transmission of rabies among free-roaming dogs is facilitated by their growing population. In 2016, Bhutan had a total dog population of 119,624 (71,245 owned dogs and 48,379 free-roaming dogs) (7). Currently, wildlife are not considered to be a key reservoir for rabies in Bhutan. Based on the epidemiology of rabies outbreaks, Bhutan is demarcated into rabies high-risk and low-risk zones (8).

**Figure 1:**
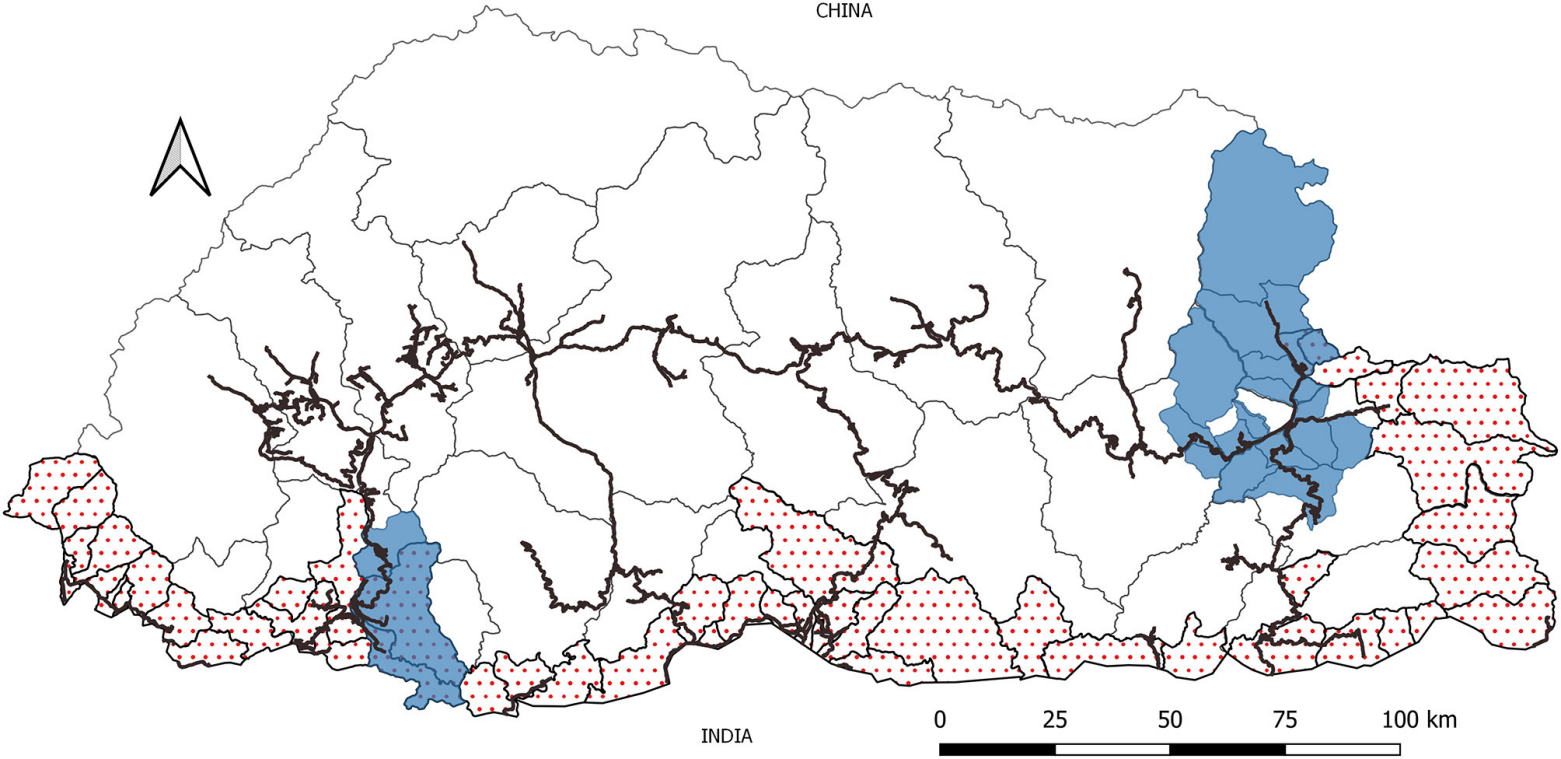
Map of Bhutan showing the rabies high-risk zone (dotted area), the rabies low-risk zone (plain areas), and the network of highways connecting high-risk zone to the low-risk zone. The areas under blue shade are sub-districts that experienced outbreaks in 2005 (East) and 2008 (South West).

The entire belt of the southern and eastern region that report rabies outbreaks are considered as rabies “high-risk zone,” while the northern and central regions, from where rabies has not been reported since 1991 are considered to be the rabies “low-risk zone” (Figure 1). Since rabies has not been reported in animals and in humans for more than two decades in the rabies low-risk zone, one of the objectives of the government is to achieve freedom from dog-mediated rabies by zone. Control measures, such as mass dog vaccination and dog population management, through animal birth control, regulation of movment of animals through pre-travel check-up and health certification, and rabies surveillance are put in place to ensure that disease is not translocated along with the movement of animals. In 2009, as an approach to manage the growing dog population and control rabies transmission, the government of Bhutan in collaboration with an US-based NGO, Humane Society Internationale (HSI) initiated the Catch, Neuter, Vaccinate and Release (CNVR) program. So far, about 105,000 dogs and cats have been neutered and vaccinated against rabies through this program (9). The CNVR program is carried out annually throughout the country by the respective district livestock sectors. In addition, in the rabies endemic areas of Bhutan, mass dog vaccination is carried out annually to create an immune buffer along the border. Furthermore, besides the free clinical and vaccination services provided to pet owners through the network of animal health facilities across the country, pets are provided free rabies vaccinations through vaccination campaigns organized during World Rabies Day. Although no recent study has been conducted to estimate the vaccination coverage in the free ranging dog population, a mark re-sight study conducted by Tenzin et al. (10) estimated average vaccination coverage of 57% in free-roaming dogs in two of the larger southern towns of Bhutan, Gelephu (56%), and Phuentsholing (58%).

Despite having these measures in place a number of factors, such as the increasing dog population (stray and pet), anthropogenic factors, such as increasing human settlements, increased dog density along the national highways, more animal/pet transportation both legally and illegally, and the complex and changing human-pet relationship, increase the potential for rabies reintroduction from high-risk zones, and from neighboring rabies endemic countries, into rabies low-risk zones of Bhutan (11). Furthermore, with the increasing incidence of incursions in the east and the recent case of rabies in an apparently healthy puppy illegally imported from India highlights the need to understand the likelihood of similar introduction and incursions in future (12). In this study, we assessed the risk of rabies reintroduction into low-risk areas of Bhutan using a qualitative risk assessment method. The findings from this study can inform risk managers about the risk of rabies reintroduction into the low-risk zones of Bhutan and identify the most effective risk mitigation options.

## Materials and Methods

### Risk Assessment Methodology

**Figure 2:**
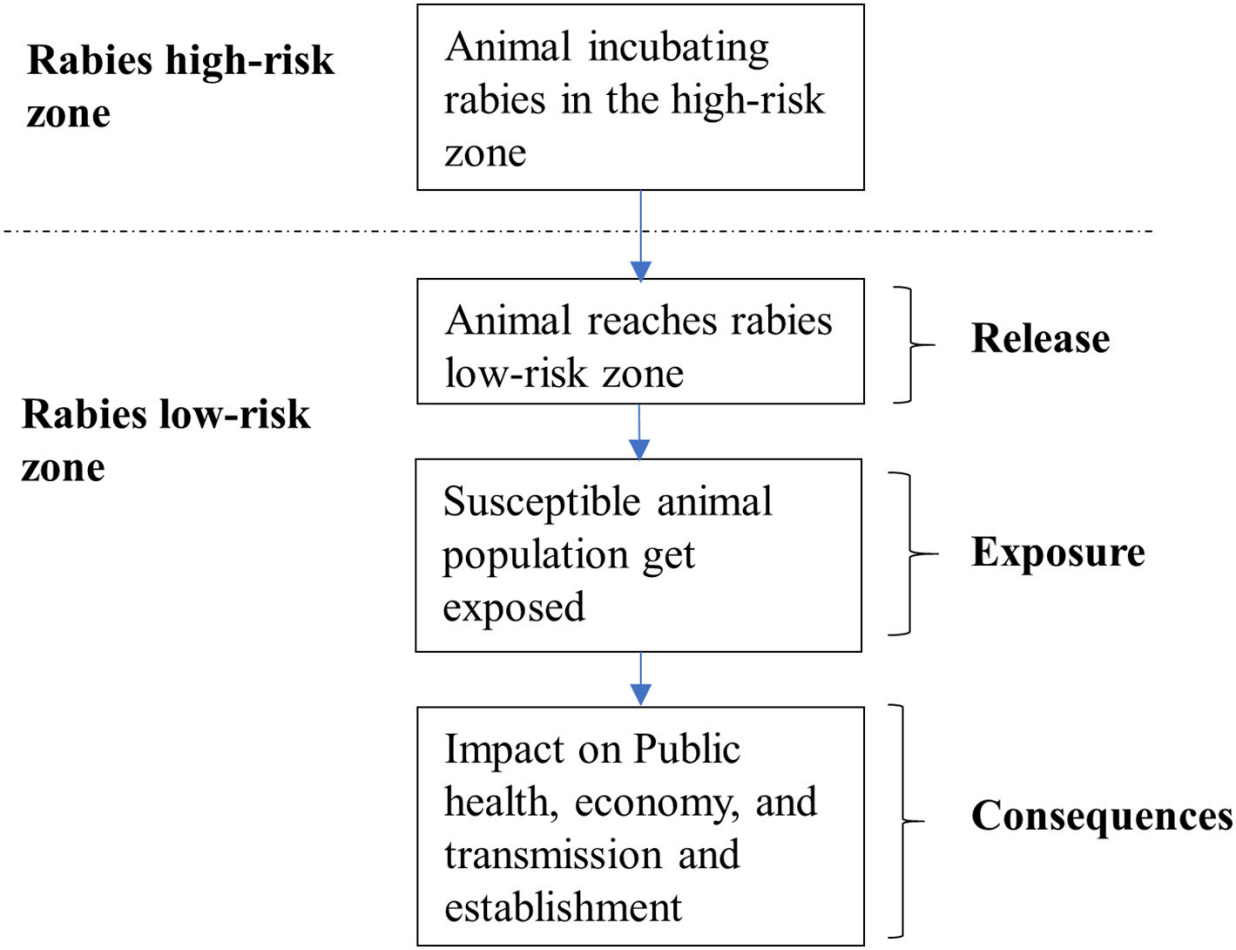
Schematic flow chart showing the events considered in each risk pathway.

A qualitative risk analysis methodology developed by the World Organization for Animal Health (OIE) for the import of animals and animal products was adopted for this study (13). Risk assessment is a component of risk analysis and consists of hazard identification, entry assessment, exposure assessment, consequence assessment, and risk estimation. For this assessment, “entry” corresponded to the entry of the hazard (i.e., rabies virus) into a rabies low-risk zone through movement of animals (dogs, cattle) incubating rabies virus. Exposure corresponded to contact and transmission resulting from the interaction between infected animals (infectious stage) and susceptible populations in the rabieslow-risk zone. The consequences of rabies reintroduction into rabies-low risk areas were assessed based on the potential for disease establishment and the likely economic and public health impacts. We used data from published literature to estimate the magnitude of the consequence of rabies reintroduction. The framework of risk assessment for this analysis is provided in Figure 2. Three most likely risk pathways, namely the stray dog, the pet dog, and cattle-mediated pathways, were considered. For the purpose of this study, those free-roaming dogs that did not have an owner and fed on community food-leftovers and waste were considered to be stray while dogs that were fed, cared for and owned/claimed to be owned by a household were considered to be pets. Subsequently, the risk of rabies reintroduction through these pathways was assessed under two scenarios, assuming that, (1) no risk mitigation measures were in place and (2) the current risk mitigation measures were in place. The mitigation measures considered in this assessment included the activities undertaken by the Department of Livestock to control rabies in the rabies high-risk zones, such as mass dog vaccination, animal birth control program, public awareness and surveillance. Further, the aspects of movement control regulations, such as a pre-travel health checkup and regulatory health certification along national highways, implemented by the Bhutan Agriculture and Food Regulatory Authority (BAFRA), were considered.

The probability of rabies transmission (i.e., rabies virus entry and exposure) occuring along each risk pathway were derived by combining the probalities of each event occurring. For example, the probability of rabies virus entry through the stray dog pathway was derived by combining the probabilities of factors, such as the prevalence of rabies in stray dogs in the rabies high-risk zone, movement of stray dogs from the high-risk to low risk/rabies free zone, likely exposure of susceptible animals and the potential for sustained dog-to-dog transmission of the disease. The risk factors assessed were based on the theoretical relevance to each pathway. The qualitative probability scales used for this assessment was adapted from Roche et al. (14) (Table 1).

**Table 1:** Qualitative probability scales with definitions used for assigning the probability to any factor or event in this assessment.

**Likelihood scale**	**Definition**
Negligible	Likelihood of an event occurring is so rare that it does not merit consideration
Extremely low	Likelihood of an event occurring is extremely rare but cannot be excluded
Very low	Likelihood of an event occurring is rare but does occur
Low	Likelihood of an event occurring is occasional
Medium	Likelihood of an event occurring is regular
High	Likelihood of an event occurring is very often

The final estimate of risk for each pathway was derived by combining the probabilities of each event occuring, i.e., entry and exposure and the magnitude of consequences due to the introduction of rabies. The combination matrix described by Dufour et al. (15) was used. This matrix accounts for the conditional nature of probability (Table 2).

**Table 2:** Combination matrix used to combine two probabilities.

**Probability**	**Negligible**	**Extremely low**	**Very low**	**Low**	**Medium**	**High**
Negligible	Negligible	Negligible	Negligible	Negligible	Negligible	Negligible
Extremely low	Negligible	Extremely low	Extremely low	Extremely low	Extremely low	Extremely low
Very low	Negligible	Extremely low	Very low	Very low	Very low	Very low
Low	Negligible	Extremely low	Very low	Low	Low	Low
Medium	Negligible	Extremely low	Very low	Low	Medium	Medium
High	Negligible	Extremely low	Very low	Low	Medium	High

*Concept adapted from Dufour et al. (15) when combining two probabilities, the resulting probability is not greater than the lower probability scale of the two*.

### Data Sources

The theoretical probability of each event/factor occurring and influencing the final risk estimate was determined using published data from the literature as proposed by Zepeda-Sein (16). Where gaps in knowledge existed, we used expert opinion. The uncertainty associated with assigning a probability was expressed in qualitative terms as described in Supplementary Table 1. The expert's evaluation of uncertainty was elicited using the guidelines adapted from Roche et al. (14) (Supplementary Table 2).

### Expert Opinion

#### Selection of Experts

To ensure that the outcome of this risk assessment was relevant to the context of the rabies control program in Bhutan, local experts working under the Department of Livestock (DoL) and Bhutan Agriculture and Food Regulatory Authority of Bhutan (BAFRA) were consulted. Initially identified due to their familiarity with the disease, and the current risk mitigation measures in Bhutan, two experts were selected from the National Center for Animal Health (NCAH). Through these experts, 15 other experts were selected from across the country. Thirteen experts, ten working under the DoL and three under BAFRA, participated in a 2-days expert opinion elicitation workshop. Four experts could not participate because of a prior commitment.

#### Ethical Statement

The Conjoint Faculties Research Ethics Board (CFREB), University of Calgary, Canada approved the study protocol (Approval number REB16-1945). An informed consent was sought from the experts before their participation.

#### Expert Opinion Elicitation

The two-stage modified Delphi technique was used for eliciting expert opinion as described by Roche et al. (14). A questionnaire containing both open ended and closed questions was used to collect the experts' opinions. The first section of the questionnaire included questions related to the source of rabies and the factors contributing to rabies outbreaks in dogs and cattle in the rabies high-risk zone. The second section comprised questions eliciting the qualitative probabilities and uncertainties determining the entry of rabies virus, exposure of susceptible populations in the rabies low-risk zone and the consequences resulting from a rabies incursion.

Two rounds of elicitation (stage 1 and stage 2) were carried out before and after an experts' workshop. In the first round, the questionnaire was emailed to the experts. Once experts had sent back the questionnaire, a preliminary analysis was carried out. Following the first round of elicitation, a 2 days workshop was conducted. The workshop was facilitated by an experienced moderator. On the first day of the workshop, the experts were briefed on the background and objectives of the workshop and an overview was provided on the broader project. Then they were introduced to the basic concept of risk assessment and the use of expert opinion in the analysis. The questions used for soliciting expert opinion during the first round of elicitation were further explained to the experts, to ensure that they had a uniform understanding of the terms used. On the second day, the preliminary results of the first round of elicitation was presented and a group discussion was facilitated.

After the workshop, the same questionnaire was sent out to the experts. The opinion elicited during the second round, and relevant to the final assessment, was used (Data Sheet 2). The two-stage modified Delphi technique was used to exploit the advantage of preserving the anonymity and independence of the experts and exploiting the benefits of group interactions (14, 17, 18).

#### Combining Expert Opinion

The expert opinions were combined as described by Gale et al. (19). For a single factor, there were thirteen qualitative probability estimates provided by the thirteen experts. These thirteen qualitative probability scales were combined, and the median scale thus derived was used for the final analysis. The median was used as it is easy to derive and is a robust measure of central tendency in the Delphi process (20, 21). The number of factors considered for each event in the pathways are listed in Tables 3–**6**. With respect to uncertainties, while combining the probabilities of entry and exposure, we considered the highest uncertainty scale along the risk pathway unless the *n* + 1 step had a probability score of negligible with low uncertainty as described by Crotta et al. (25).

**Table 3:** Estimates of rabies virus entry through three risk pathways (with no risk mitigation measures in place).

**Entry of rabies virus**	**Probability**	**Uncertainty**	**Evidence**	**Estimate (Uncertainty)**
**STRAY DOG PATHWAY**				
- Probability determined by prevalence of rabies in stray dogs	Medium	Low	(8)	Medium (Low)
- Probability determined by stray dog movement	Medium	Low	(4, 5, 22, 23)	
- Probability determined by sustained transmission	Medium	Low	(4, 5, 23)	
**PET DOG PATHWAY**				
- Probability determined by prevalence of rabies in pet dogs in the endemic areas	Low	Low	Expert opinion	Low (Low)
- Probability determined by frequency of pet dogs traveled to the rabies-free areas	Low	Low	Expert opinion	
**CATTLE PATHWAY**				
- Probability determined by prevalence of rabies in cattle in the endemic areas	Low	Low	(8, 24) and Expert opinion	Low (Low)
- Probability determined by the number of cattle transported	Low	Low	Expert opinion	

#### Sensitivity Analysis

We assessed the effect of the experts' uncertainty on our estimates. The uncertainty-weighted median was derived based on the uncertainty level provided by the experts as described Gale et al. (19). We used three scales to elicit uncertainties associated with the experts' opinions as described in Supplementary Table 2. If an expert scaled the probability of a factor as “medium” (i.e., 4 on a numeric scale) with an uncertainty level of “low” (i.e., 3 on the numeric score) the particular expert would contribute 3 “medium”/“4” to the overall probability assessment of the factor. Similarly, if an expert scaled probability of a factor as “low” (i.e., 3 on a numeric scale) with an uncertainty level of “high” (i.e., 1 on the numeric score), the expert would contribute only one “low”/“3” to the overall probability assessment of the factor. The more certain an expert is about the probability of a factor, the more probability scale for the factor would the expert contribute and vice versa. Subsequently, the median probability scale was derived and used for analysis.

## Results

### Rabies Virus Entry and Exposure Probabilities

The probability of rabies virus entry through the stray dog pathway under the scenario with no risk mitigation measures was higher than the scenario with current mitigation measures in place (medium with a low level of uncertainty vs. very low with a medium level of uncertainty). Similarly, the probability of virus entry through cattle under the scenario with no risk mitigation measures was higher than the scenario with the current mitigation measures in place (low probability with a low level of uncertainty vs. very low with a low level of uncertainty). The probability of rabies virus entry and the level of uncertainty did not change for the pet dog pathway (Tables 4–6).

**Table 4:** Estimates of entry for the three pathways (with the current risk mitigation measures).

**Entry of rabies virus**	**Probability**	**Uncertainty**	**Evidence**	**Estimate (Uncertainty)**
**STRAY DOG PATHWAY**				
-Probability determined by prevalence of rabies in stray dogs	Medium	Low	Government report	Very low (Medium)
-Probability determined by tendency of stray dogs to move	Medium	Medium	(4, 5, 22, 23)	
-Probability of current passive veterinary surveillance system not detecting rabid dogs in the rabies endemic areas^*^	Very low	Low	Expert opinion	
-Probability of public not reporting cases (public awareness)^*^	Very low	Low	Expert opinion	
**PET DOG PATHWAY**				
-Determined by prevalence of rabies in pet dogs in the endemic areas	Low	Low	Expert opinion	low (Low)
-Determined by frequency of pet dogs traveled to the rabies low-risk zone	Low	Low	Expert opinion	
-Probability of pet owners not being aware of pre-travel check-up^*^	Low	Low	Expert opinion	
-Probability of highway check posts not detecting the pets traveled without pre-check-up^*^	Medium	Low	Expert opinion	
**CATTLE PATHWAY**				
- Determined by prevalence of rabies in cattle in the endemic areas of Bhutan	Low	Low	(26), TAD info database	Low (Low)
-Determined by the number of cattle transported	Low	Low	Expert opinion	
-Probability of cattle not undergoing pre-travel check-up^*^	Low	Low	Expert opinion	

* *Probability of entry resulting due to ineffectiveness of current mitigation measures*.

**Table 5:** Estimates of exposure probabilities in susceptible populations (with no risk mitigation measures in place).

**Exposure**	**Probability**	**Uncertainty**	**Evidence**	**Estimate (Uncertainty)**
Stray dog pathway	Medium	Low	(4, 5, 27, 28)	Medium (Low)
Pet dog pathway	Low	Low	(12, 29–31)	Low (Low)
Cattle pathway	Very low	Medium	(32–35)	Very low (Medium)

**Table 6:** Estimates of exposure probabilities in susceptible populations (with current risk mitigation measures in place).

**Exposure of susceptible population**	**Probability**	**Uncertainty**	**Supporting evidence**	**Estimate (Uncertainty)**
**STRAY DOG MEDIATED PATHWAY**				
- Probability determined by density of susceptible population	Medium	Low	(4, 5, 7)	Low (Low)
- Probability of public in the rabies low-risk zone not reporting on seeing rabid dog (public awareness)^*^	Low	Low	Expert opinion	
- Probability of current passive veterinary surveillance not detecting rabid stray dog^*^	Medium	Low	Expert opinion	
**PET MEDIATED PATHWAY**				
- Probability determined by density of susceptible population	Medium	Low	(29, 30)	Low (Low)
- Probability of public in the rabies low-risk zone not reporting on seeing rabid pet dog (public awareness)^*^	Low	Low	Expert opinion	
- Probability of pet intermingling with other animals determined by pet-owning practice^*^	Medium	Low	Expert opinion	
**CATTLE MEDIATED PATHWAY**				
- Probability of cattle owner not reporting suspected rabies in cattle^*^	Very low	Low	Expert opinion (31, 35)	Very low (Low)

* *Ineffectiveness of current mitigation measures contributing to the probability of exposure in the susceptible populations. Although these mitigation measures are less likely to prevent the first exposure, they can be helpful in widespread subsequent exposures*.

The probability of susceptible populations in the rabies low risk zone exposing to the rabies virus through the stray dog pathway was higher when the current mitigation measures were not accounted (medium with a low level of uncertainty vs. low with a low level of uncertainty). The probability of exposure through pet dog pathway remained same for both the scenarios (low with a low level of uncertainty). Similarly, except for the level of uncertainty, the probability of exposure through cattle pathway remained same for both the scenarios (very low with a medium level of uncertainty vs. very low with a low level of uncertainty) (Tables 4–6).

### Consequences

Assessing the likelihood of rabies establishment in the reservoir population, and the impact on the economy and public health based on the literature evidence, the magnitude of consequences for the stray dog, pet dog, and the cattle pathways were scaled high, medium, and low, respectively.

### Risk Estimation

#### Under the Scenario With No Mitigation Measures

**Figure 3:**
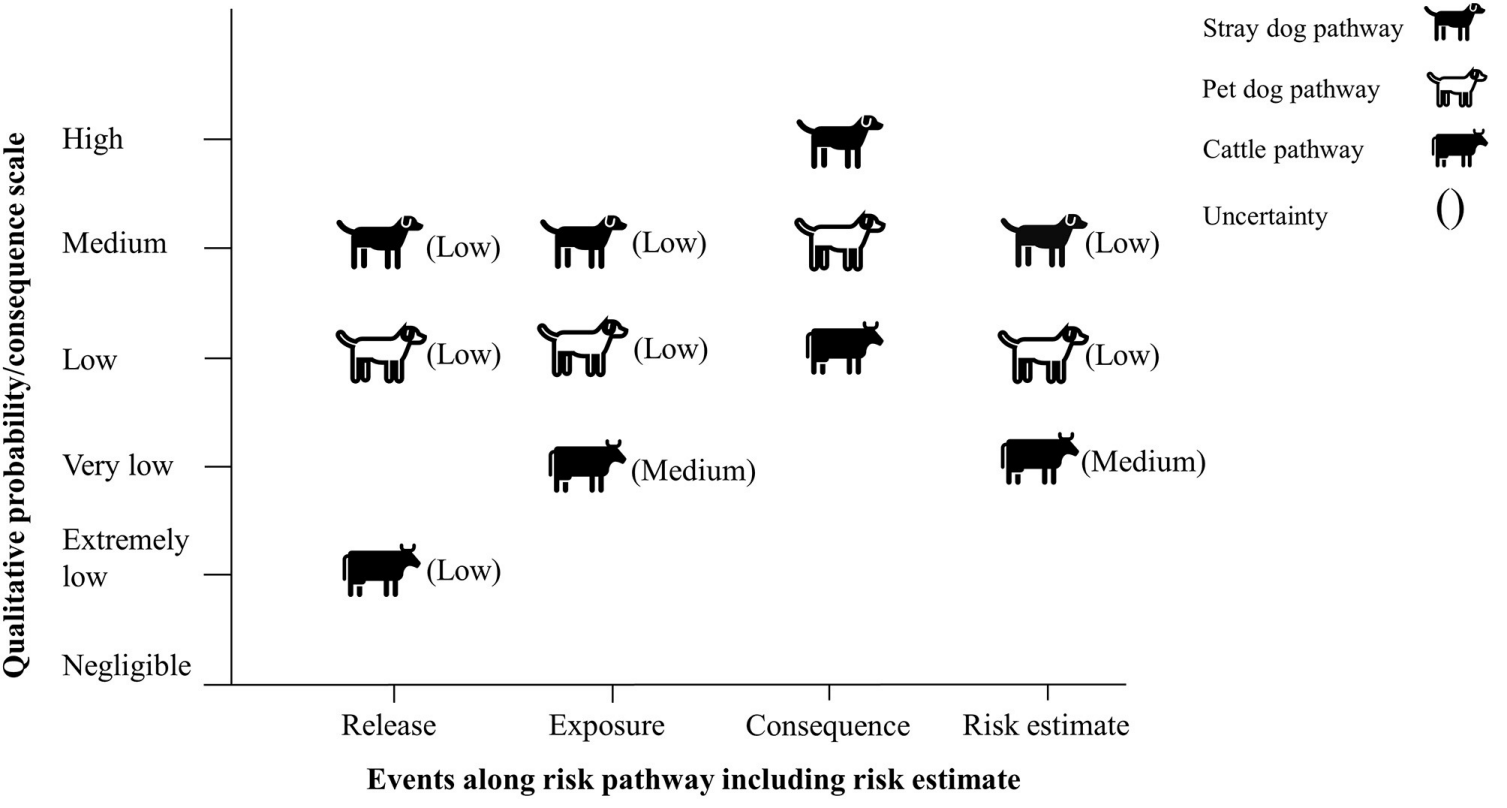
Final risk estimate obtained by combining probability of entry × probability of exposure × magnitude of consequences (no mitigation measures).

The risk of rabies reintroduction was estimated to be medium for the stray dog pathway with a low level of uncertainty, low for the pet dog pathway with a low level of uncertainty and very low for the cattle pathway with a medium level of uncertainty (Figure 3).

#### Under the Scenario With Current Mitigation Measure

**Figure 4:**
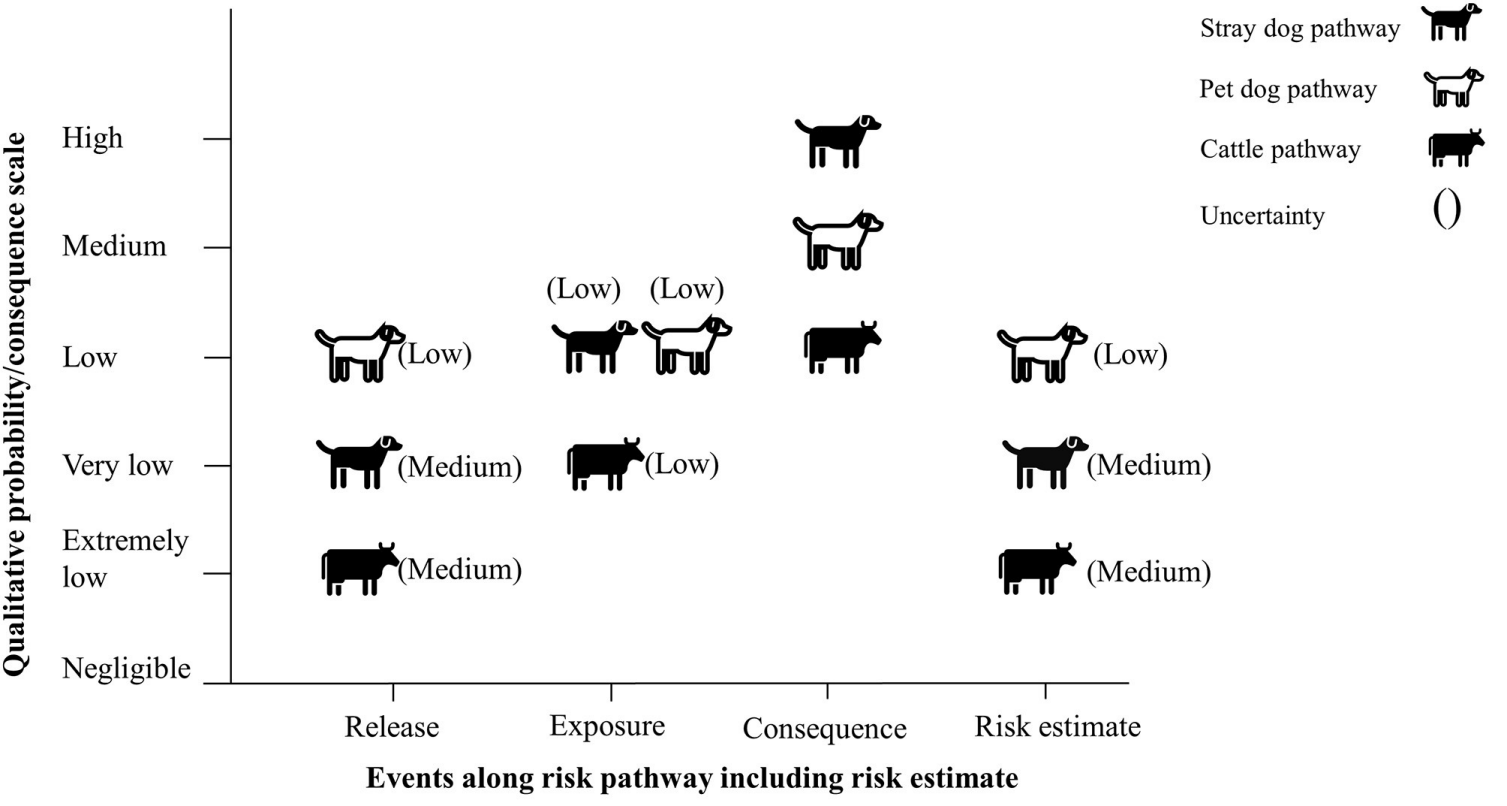
Final risk estimate obtained by combining probability of entry × probability of exposure × magnitude of consequences (with current mitigation measures in place).

The risk of rabies reintroduction into low risk zones was estimated to be very low for the stray dog pathway with a medium level of uncertainty, low for the pet dog pathway with a low level of uncertainty and extremely low for the cattle pathway with a medium level of uncertainty (Figure 4).

### Sensitivity Analysis

Based on the sensitivity analysis performed using the probability estimates derived by considering the experts' uncertainties (uncertainty-weighted median), no changes were observed in the probability estimates of rabies virus release and exposure for all three pathways.

## Discussion

In this study, we conducted a qualitative assessment of the risk of rabies reintroduction into rabies low-risk zones of Bhutan from rabies high-risk zones through the stray dog, pet dog, and cattle pathways. In Bhutan, dogs are the primary rabies reservoir, therefore two pathways (stray and pet dogs) were considered for this assesment. In the endemic areas of Bhutan, cattle are the most commonly affected livestock species (36, 37). Due to the government's policy of achieving self-sufficiency in livestock produce, in addition to mobilization of cattle within the country, a large number of cattle have been imported from other countries posing risk of disease introduction and spread, inlcuding rabies. For these reasons, the cattle pathway was included in this assessment. The assessment was conducted under two scenarios: (1) with no existing risk mitigation measures in place, and (2) with existing risk mitigation measures in the country for rabies. The risk of rabies reintroduction through all the pathways considered was above negligible under both the scenarios. The inclusion of an assessment with risk mitigation measures in place allowed us to assess the impact of current rabies control measures on the final risk estimates. Such exercises can be helpful in informing decision makers about the importance of investment in on-going risk mitigation measures.

The risk estimates were highest for the stray dog pathway followed by the pet dog and cattle pathways when the effect of current mitigation measures was not accounted for. The risk estimates for the stray dog pathway being the highest are supported by observations from previous rabies incursions in the east and south-western areas of Bhutan. Major outbreaks of dog-mediated rabies in rabies low risk zones of Bhutan occurred because of the movement of stray dogs from rabies high-risk zones (4, 5) and from rabies endemic areas in neighboring states of India. Factors, such as the growing stray dog population associated with increasing human settlement along the highways connecting high-risk and low risk zone may enable survival and interaction among dogs, thus continuing the rabies transmission chain.

The decrease in the risk estimate for the stray dog pathway when accounting for current risk mitigation measures in place was not surprising. This can be related to the enhanced dog population management and rabies control program that is currently being implemented throughout the country. Records show that more than 102,316 dogs and cats (stray and owned) have been sterilized and vaccinated in the country since 2009. Furthermore, the government has also strengthened strategies to disseminate rabies awareness education and enhanced movement control regulations for animals from both outside and within the country.

The OIE import risk analysis methodology requires a comprehensive assessment of the consequences of a disease incursion into disease-free areas considering the impact on the economy, on public health, and on the environment. A thorough assessment of consequences, therefore, requires a One Health approach engaging a wide range of expertise including veterinarians, epidemiologists, disease ecologists, economists, public health practitioners and other relevant expertise (13). For this study, given that there is adequate information about the public health and economic impacts of rabies, we used available literature to estimate the magnitude of the consequences of rabies reintroduction.

The high magnitude of consequences for stray dog pathway can be linked to the higher likelihood of rabies establishment in the reservoir population. As in the case of rabies introduction in the Flores Island, Indonesia in 1997 (38, 39), the high likelihood of rabies establishment in rabies low-risk zone is associated with low level of vaccination coverage in the free-roaming dogs, low awareness level among the general public to report sighting of rabid dogs early and lack of active surveillance in place. The rabies establishment in the dog population would result in huge public health and economic impact for both government and community. For example, the Asian and African countries that report the highest disease burden from canine rabies are mostly due to free-roaming dogs which are responsible for several thousands of human mortalities and significant economic losses (1, 40). In addition, outbreaks in free-roaming dogs lead to widespread transmission with spillover infection in humans and livestock, thereby requiring implementation of large scale control measures. In 2005 and 2008, there were two outbreaks reported in the areas in the low-risk zone of Bhutan. During these outbreaks, 245 domestic livestock were also infected, and a single case of human mortality was reported (4, 5). Although the cost incurred in responding to the outbreak in 2005 was not calculated, the direct response cost for the outbreak in 2008 alone was estimated to be about Nu. 2.75 Million (≈US $59,923; 1 US $ = Nu. 46), which included the value for the lost livestock, post-exposure prophylaxis for humans, the cost of vaccinating, impounding and culling dogs, and organizing awareness campaigns and cost for the rapid response team (4, 5).

Whereas, the lower magnitude of consequences for pet dog and cattle pathway can be associated with lower likelihood of rabies establishment in the reservoir population and relatively minimal public health and economic implications. The public health risk of rabies from pet dogs that are kept in complete confinement within the home premises is limited to the family members and animal health personnel attending to the sick pet dog. A case of rabies in a pet dog transported from India was reported from Haa, a town in northern Bhutan. Because the dog was kept inside the house and was on a leash when it was taken for walks during the incubation period of infection, indirect exposure (no direct bite) resulted to only nine people requiring post-exposure prophylaxis (12). However, currently in Bhutan, a large proportion of pet owners let their dogs roam freely. A survey conducted in two major southern towns of Bhutan observed that 31% of the free-roaming dogs were “owned” (7). In a study carried out by Tenzin et al. (41), it was observed that 29% of the total animal bite victims who came to seek PEP were reported to have been bitten by dogs that had an owner. Such practice of pet ownership not only increases the likelihood of human exposure to rabid pet dogs but also increases the likelihood of rabies transmission to the free-roaming dog population and subsequent establishment of rabies endemicity.

The likelihood of rabies establishment in the susceptible dog population in the rabies-free areas is insignificant for the cattle pathway because cattle are generally considered to be a “dead-end” host. However, the economic impact of rabies in cattle (particularly at the local community level) and potential public health concerns can be substantial. For example, a loss of cattle due to rabies is a significant economic loss to a marginal cattle owner where cattle play an essential role in sustaining their livelihoods. Furthermore, people, particularly the cattle owners and animal health workers, can be exposed to rabies from cattle either by sustaining bites while handling or by abraded skin or mucous membrane contacting infectious materials. Two incidents of veterinary staff dying of rabies after contracting an infection while handling rabid cattle and small ruminants have been reported from Brazil and Iran (34, 35). Handling of sick animals, tending to minor abrasions or cut wounds, opening and examining the mouth of an animal refusing to eat are some of the common practice cattle owners engage in on their farm. Furthermore, in marginalized communities, dead cattle (irrespective of the cause of death) are commonly dressed for consumption and sale. Such practices could lead to potential exposure to rabies. There is a report from Iran of shepherds who died of rabies after dressing wounds in their sheep inflicted by a rabid wolf (35). A fatality due to rabies has also been reported from Pakistan in a butcher who skinned a calf that had died after expressing some signs associated with the neurological disorder (32).

There is uncertainty around the risks associated with the consumption of unpasteurized milk. It was reported that a lamb became infected with rabies after suckling from an experimentally infected ewe (42), and a recent study in India has demonstrated the presence of rabies viral RNA in the milk of cattle (buffaloes and cows) suspected of rabies (43). Although there is no risk of rabies from drinking pasteurized milk, the risk of contracting rabies from drinking raw milk has been cited as theoretically possible (33). Therefore, currently in most cases, people who have consumed raw milk derived from cattle that had subsequently died of rabies are provided post-exposure prophylaxis, thus increasing the cost of treatment. Annually on average, around 10% of people who receive PEP for rabies in Bhutan do so due to exposure resulting from outbreaks of rabies in cattle (41). Cases of mass exposure in humans resulting from handling rabid cattle and consuming dairy and meat products from cattle that had died of rabies have been reported from other parts of the world (33, 44). Although the risk of rabies establishment in the dog population due to transmission resulting from cattle in the areas under rabies low-risk zone is negligible, the economic and precautionary public health interventions resulting from exposure to infected cattle can still be substantial.

As described by Wieland et al. (45), using local experts in this assessment ensured that the risk assessment was relevant to the local context and the findings could be considered for policy formulation and decision making. However, it is acknowledged that the experts were in a way “custodians” of the existing rabies risk mitigation options in Bhutan. Therefore, we anticipate some bias in the expert opinions particularly regarding the effectiveness of current mitigation measures. Nevertheless, the benefits of engaging local experts outweigh the bias—if any—that may result from their engagement.

A major complication of this study was regarding the exposure assessment. The probability of exposure in dogs, domestic livestock, and humans in the rabies low-risk zone resulting from each pathway would differ depending on circumstances. Therefore, although unlikely, we assumed that the exposure from an introduced rabid animal would be the same for all the susceptible populations considered, including humans in the rabies low-risk zone. In this assessment, we consider that the risk of rabies reintroduction estimated for each pathway will be uniform across the rabies low-risk zone. However, given the differences in geographical features, road connections, human settlements, and relative distance from the rabies high-risk zone, the risk may vary from place to place within the rabies low-risk zone. Therefore, our estimates of rabies virus entry, exposure, and the overall risk may not be uniform for the entire rabies low-risk zone in Bhutan.

Although the qualitative risk assessment methodology has its limitations, especially in the definition of the qualitative probability scales, subjectivity associated in assigning probability and magnitude of consequence, and combining qualitative probabilities (46, 47), it is simple to conduct, easy to communicate, and most importantly an accepted methodology (48). Furthermore, a qualitative assessment is recommended in order to identify important chains of events and critical control points along risk pathways. This can then be used to construct robust and informed risk management programs when there is insufficient data to conduct a meaningful quantitative assessment. Nevertheless, we acknowledge the value that a quantitative risk assessment can add when sufficient data is available.

## Conclusion

From this study, we observed that the risk of rabies reintroduction through all the pathways considered in this assessment was above negligible. The risk estimate was highest for the stray dog pathway when no mitigation measures were accounted for. However, when we did account for current mitigation measures, the risk of rabies reintroduction remained above negligible for all pathways. This finding warrants enhancing public awareness and participation, especially through fostering responsible pet ownership and encouraging compliance with animal health checks and movement control regulations. The effectiveness of the current risk mitigation measures was evident as the estimates for the probabilities of rabies virus entry and exposure decreased when the mitigation measures were accounted for in the assessment. Therefore, this finding highlights the importance of maintaining and enhancing current risk mitigation measures as an important risk management option to prevent rabies reintroduction into the rabies low-risk zone of Bhutan. It is thus vital to enhance current rabies control programs (e.g., dog vaccination, awareness education, and surveillance) in the endemic areas and to strengthen the health assessment and movement control regulations for dogs and other species.

## Data Availability Statement

All datasets generated for this study are included in the article/Supplementary Material.

## Ethics Statement

The study was reviewed and approved by the Conjoint Faculties Research Ethics Board (CFREB), University of Calgary, Canada approved the study protocol (Approval REB16-1945). The patients/participants provided their written informed consent to participate in this study.

## Author Contributions

SR, SC, and DH conceived, coordinated and executed the study. TT contributed to study design and expert workshop organization. All the authors have contributed to drafting the manuscript, critically reviewing and revising it and approved the final manuscript.

## Conflict of Interest

The authors declare that the research was conducted in the absence of any commercial or financial relationships that could be construed as a potential conflict of interest.

## References

[1] Hampson K Coudeville L Lembo T Sambo M Kieffer A Attlan M. Estimating the global burden of endemic canine rabies. PLoS Negl Trop Dis. (2015) 9:e0003709. 10.1371/journal.pntd.0003709 25881058 PMC4400070

[2] Tenzin Ward MP. Review of rabies epidemiology and control in south, south east and east Asia: past, present and prospects for elimination. Zoonoses Public Health. (2012) 59:451–67. 10.1111/j.1863-2378.2012.01489.x 23180493

[3] Tenzin Namgyal J Letho S. Community-based survey during rabies outbreaks in Rangjung town, Trashigang, eastern Bhutan, 2016. BMC Infect Dis. (2017) 17:281. 10.1186/s12879-017-2393-x 28415972 PMC5393039

[4] Tenzin T Dhand NK Dorjee J Ward MP. Re-emergence of rabies in dogs and other domestic animals in eastern Bhutan, 2005–2007. Epidemiol Infect. (2010) 139:220–5. 10.1017/S0950268810001135 20492745

[5] Tenzin Sharma B Dhand NK Timsina N Ward MP. Reemergence of rabies in Chhukha District, Bhutan, 2008. Emerg Infect Dis. (2010) 16:1925–30. 10.3201/eid1612.100958 21122223 PMC3294548

[6] Owoyele GD Rabies outbreak in Thimphu, Bhutan. Bhutan J Anim Husband. (1992) 13:36–9.

[7] Rinzin K Tenzin T Robertson I. Size and demography pattern of the domestic dog population in Bhutan: implications for dog population management and disease control. Prevent Vet Med. (2016) 26:39–47. 10.1016/j.prevetmed.2016.01.030 26873612

[8] NCAH National Rabies Prevention and Control Plan. 2nd ed. NCAH (2017).

[9] Tshedup Y Engaging Residents to Curb Dog Population. Gelephu: Kuensel (2020).

[10] Tenzin T McKenzie JS Vanderstichel R Rai BD Rinzin K Tshering Y. Comparison of mark-resight methods to estimate abundance and rabies vaccination coverage of free-roaming dogs in two urban areas of south Bhutan. Prevent Vet Med. (2015) 118:436–48. 10.1016/j.prevetmed.2015.01.008 25650307

[11] Tenzin Dhand NK Ward MP. Anthropogenic and environmental risk factors for rabies occurrence in Bhutan. Prevent Vet Med. (2012) 107:21–6. 10.1016/j.prevetmed.2012.05.003 22673581

[12] Tenzin Imported Rabies Case in Pet Dog at Haa town: Investigation and Risk Assessment Report. Thimphu: National Centre for Animal Health; DoL (2016).

[13] OIE. Handbook on Import Risk Analysis for Animals and Animal Products. Volume 1: Introduction and Qualitative Risk Analysis. Paris: OIE (World Organisation for Animal Health) (2004). p. 57.

[14] Roche SE Costard S Meers J Field HE Breed AC. Assessing the risk of Nipah virus establishment in Australian flying-foxes. Epidemiol Infect. (2015) 143:2213–26. 10.1017/S0950268813003336 24580962 PMC9506974

[15] Dufour B Plée L Moutou F Boisseleau D Chartier C Durand B. A qualitative risk assessment methodology for scientific expert panels. Rev Sci Tech. (2011) 30:673–81. 10.20506/rst.30.3.2063 22435181

[16] Zepeda-Sein C Méthodes d'évaluation des risqueszoosanitaires lors des échanges internationaux. In: Séminairesur la Sécurité Zoosanitaire des échanges dans les Caraïbes, Port of Spain, Trinidad, and Tobago. Paris: World Organisation for Animal Health (OIE) (1998). p. 2–17.

[17] Mosleh A Bier VM Apostolakis G A critique of current practice for the use of expert opinions in probabilistic risk assessment. Reliabil Eng Syst Safety. (1988) 20:63–85. 10.1016/0951-8320(88)90006-3

[18] Gustafson DH Shukla RK Delbecq A Walster GW A comparative study of differences in subjective likelihood estimates made by individuals, interacting groups, Delphi groups, and nominal groups. Organ Behav Hum Decis Perform. (1973) 9:280–91. 10.1016/0030-5073(73)90052-4

[19] Gale P Brouwer A Ramnial V Kelly L Kosmider R Fooks A. Assessing the impact of climate change on vector-borne viruses in the EU through the elicitation of expert opinion. Epidemiol Infect. (2010) 138:214–25. 10.1017/S0950268809990367 19580695

[20] Larréché J-C Moinpour R Managerial judgment in marketing: the concept of expertise. J Market Res. (1983) 20:110–21. 10.1177/002224378302000202

[21] Scholz RW Hansmann R. Combining experts' risk judgments on technology performance of phytoremediation: self-confidence ratings, averaging procedures, and formative consensus building. Risk Anal. (2007) 27:225–40. 10.1111/j.1539-6924.2006.00871.x 17362411

[22] Pal SK Ghosh B Roy S Dispersal behaviour of free-ranging dogs (*Canis familiaris*) in relation to age, sex, season and dispersal distance. Appl Anim Behav Sci. (1998) 61:123–32. 10.1016/S0168-1591(98)00185-3

[23] Waltner-Toews D Maryono A Akoso BT Wisynu S Unruh DHA An epidemic of canine rabies in Central Java, Indonesia. Prevent Vet Med. (1990) 8:295–303. 10.1016/0167-5877(90)90087-X

[24] NCAH Status of Notifiable Animal Diseases in Bhutan. Thimphu: National Centre for Animal Health (2015).

[25] Crotta M Ferrari N Guitian J Qualitative risk assessment of introduction of *Anisakid larvae* in Atlantic salmon (*Salmo salar*) farms and commercialization of products infected with viable nematodes. Food Control. (2016) 69:275–84. 10.1016/j.foodcont.2016.04.058

[26] NCAH Status of Notifiable Animal Diseases in Bhutan. Thimphu: National Centre for Animal Health (2014).

[27] Putra AAG Hampson K Girardi J Hiby E Knobel D Mardiana IW. Response to a rabies epidemic, Bali, Indonesia, 2008–2011. Emerg Infect Dis. (2013) 19:648–51. 10.3201/eid1904.120380 23632033 PMC3647408

[28] IRIN Record Rabies Outbreak Kills 93 Children. (2009). Available online at: http://www.irinnews.org/news/2009/03/11/record-rabies-outbreak-kills-93-children (accessed September 13, 2017).

[29] Castrodale L Walker V Baldwin J Hofmann J Hanlon C. Rabies in a puppy imported from India to the USA, March 2007. Zoonoses Public Health. (2008) 55:427–30. 10.1111/j.1863-2378.2008.01107.x 18833596

[30] Ribadeau-Dumas F Cliquet F Gautret P Robardet E Pen Cl Bourhy H. Travel-associated rabies in pets and residual rabies risk, western Europe. Emerg Infect Dis. (2016) 22:1268–71. 10.3201/eid2207.151733 27314463 PMC4918150

[31] Martin RJ Schnurrenberger PR Walker JF. Exposure to rabies–an occupational hazard for veterinarians. Can Vet J. (1982) 23:317–23. 17422197 PMC1790226

[32] Tariq WUZ Shafi MS Jamal S Ahmad M. Rabies in man handling infected calf. Lancet. (1991). 337:1224. 10.1016/0140-6736(91)92895-9 1673761

[33] CDC Mass Treatment of Humans Who Drank Unpasteurized Milk from Rabid Cows–Massachusetts, 1996–1998. (1999). Available online at: http://www.cdc.gov/mmwr/preview/mmwrhtml/00056759.htm (accessed October 25, 2016). 10220255

[34] Simani S Fayaz A Rahimi P Eslami N Howeizi N Biglari P. Six fatal cases of classical rabies virus without biting incidents, Iran 1990–2010. J Clin Virol. (2012) 54:251–4. 10.1016/j.jcv.2012.03.009 22554714

[35] Brito MGd Chamone TL Silva FJd Wada MY Miranda ABd Castilho JG. Antemortem diagnosis of human rabies in a veterinarian infected when handling a herbivore in Minas Gerais, Brazil. Rev Inst Med Trop Sáo Paulo. (2011) 53:39–44. 10.1590/S0036-46652011000100007 21412618

[36] Rinchen S Tenzin T Hall D van der Meer F Sharma B Dukpa K. A community-based knowledge, attitude, and practice survey on rabies among cattle owners in selected areas of Bhutan. PLoS Neglect Trop Dis. (2019) 13:e0007305. 10.1371/journal.pntd.0007305 30933984 PMC6459539

[37] Tenzin Ward MP Dhand NK Epidemiology of rabies in Bhutan: geographical information system-based analysis. In: Fooks AR Müller T, editors. Compendium of the OIE Global Conference on Rabies Control, Incheon-Seoul, Republic of Korea, 7-9 September 2011 Towards sustainable prevention at the source. Paris: OIE (World Organisation for Animal Health) (2012). p. 67–73.

[38] Wera E Velthuis AG Geong M Hogeveen H. Costs of rabies control: an economic calculation method applied to Flores Island. PLoS ONE. (2013) 8:e83654. 10.1371/journal.pone.0083654 24386244 PMC3873960

[39] Windiyaningsih C Wilde H Meslin FX Suroso T Widarso H. The rabies epidemic on Flores Island, Indonesia (1998–2003). J Med Assoc Thailand. (2004) 87:1389–93. 15825719

[40] Knobel DL Cleaveland S Coleman PG Févre EM Meltzer MI Miranda MEG. Re-evaluating the burden of rabies in Africa and Asia. Bull World Health Organ. (2005) 83:360–8. 10.1590/S0042-96862005000500012 15976877 PMC2626230

[41] Tenzin Dhand NK Ward MP. Human rabies post exposure prophylaxis in Bhutan, 2005-2008: trends and risk factors. Vaccine. (2011) 29:4094–101. 10.1016/j.vaccine.2011.03.106 21497633

[42] Afshar A. Review of not-bite transimission of rabies virus infection. Br Vet J. (1979) 135:142–8. 10.1016/S0007-1935(17)32935-4 427522

[43] Dandale M Singh CK Sandhu BS Bansal K Sood NK Intravitam diagnosis of rabies from milk: comparison of nested RT-PCR with TaqMan real time PCR. IOSR J Agric Vet Sci. (2012) 1:12–5. 10.9790/2380-0111215

[44] ProMed-mail. Rabies–Nepal: Kanchipur, Canine, Cow Milk, Human Exposure Suspected. (2017). Available online at: http://www.promedmail.org/post/20171008.5366422 (accessed November 12, 2017).

[45] Wieland B Dhollander S Salman M Koenen F. Qualitative risk assessment in a data-scarce environment: a model to assess the impact of control measures on spread of African Swine Fever. Prevent Vet Med. (2011) 99:4–14. 10.1016/j.prevetmed.2011.01.001 21292336

[46] Peeler EJ Reese RA Thrush MA. Animal disease import risk analysis–a review of current methods and practice. Transbound Emerg Dis. (2015) 62:480–90. 10.1111/tbed.12180 24237667

[47] Cox LA Jr. Babayev D Huber W. Some limitations of qualitative risk rating systems. Risk Anal. (2005) 25:651–62. 10.1111/j.1539-6924.2005.00615.x 16022697

[48] Moutou F Dufour B Ivanov Y. A qualitative assessment of the risk of introducing foot and mouth disease into Russia and Europe from Georgia, Armenia and Azerbaijan. Rev Sci Tech Int Epiz. (2001) 20:723–30. 10.20506/rst.20.3.1307 11732414

