# Supplementary material for: A Qualitative Risk Assessment of Rabies Reintroduction Into the Rabies Low-Risk Zone of Bhutan
Source: Front Vet Sci. 2020 Jul 14;7:366. doi: 10.3389/fvets.2020.00366 (PMC7381201; doi:10.3389/fvets.2020.00366)
Supplement: Supplementary file 1 [file Data_Sheet_1.DOCX]

Supplementary Material

# Supplementary Figures and Tables

Table 1 Qualitative description of uncertainty scales used for the qualitative risk assessment of rabies reintroduction into the rabies low-risk zone of Bhutan. This table was adapted from Roche *et al.* (2015)

| **Uncertainty scale** | **Description** |
| --- | --- |
| High | Scarce or no data available; evidence is not provided in references but rather in unpublished reports, based on observations, or personal communications; authors report conclusions that vary considerably between them |
| Medium | Some but no complete data available; evidence provided in small number of references; authors report conclusions that vary from one another |
| Low | Solid and complete data available; strong evidence provided in multiple references; authors report similar conclusions |

Table 2 Table used to guide experts in assigning their level of uncertainty while assigning a probability of a factor or an event. This table was adapted from Roche et al. (2015)

| **Uncertainty scale** | **Definition** |
| --- | --- |
| **High** | Represents that the expert is completely uncertain about his opinion. The expert has not observed or experienced such events occurring |
| **Medium** | Represents that the expert has a low level of certainty about his opinion. The expert has observed or experienced contradicting events and not very certain if occurrence of an event follows similar trend or is predictable |
| **Low** | Represents that the expert is very certain about his opinion. The expert has observed or experienced events occurring and is very certain about its occurrence |
